# Heparanase—A single protein with multiple enzymatic and nonenzymatic functions

**DOI:** 10.1002/pgr2.6

**Published:** 2023-07-09

**Authors:** Israel Vlodavsky, Yasmin Kayal, Maram Hilwi, Soaad Soboh, Ralph D. Sanderson, Neta Ilan

**Affiliations:** ^1^ Technion Integrated Cancer Center, Technion Rappaport Faculty of Medicine Haifa Israel; ^2^ Department of Pathology University of Alabama at Birmingham Birmingham Alabama USA

**Keywords:** heparanase, heparanase 2, heparan sulfate, signal transduction

## Abstract

Heparanase (Hpa1) is expressed by tumor cells and cells of the tumor microenvironment and functions extracellularly to remodel the extracellular matrix (ECM) and regulate the bioavailability of ECM‐bound factors, augmenting, among other effects, gene transcription, autophagy, exosome formation, and heparan sulfate (HS) turnover. Much of the impact of heparanase on tumor progression is related to its function in mediating tumor‐host crosstalk, priming the tumor microenvironment to better support tumor growth, metastasis, and chemoresistance. The enzyme appears to fulfill some normal functions associated, for example, with vesicular traffic, lysosomal‐based secretion, autophagy, HS turnover, and gene transcription. It activates cells of the innate immune system, promotes the formation of exosomes and autophagosomes, and stimulates signal transduction pathways via enzymatic and nonenzymatic activities. These effects dynamically impact multiple regulatory pathways that together drive tumor growth, dissemination, and drug resistance as well as inflammatory responses. The emerging premise is that heparanase expressed by tumor cells, immune cells, endothelial cells, and other cells of the tumor microenvironment is a key regulator of the aggressive phenotype of cancer, an important contributor to the poor outcome of cancer patients and a valid target for therapy. So far, however, antiheparanase‐based therapy has not been implemented in the clinic. Unlike heparanase, heparanase‐2 (Hpa2), a close homolog of heparanase (Hpa1), does not undergo proteolytic processing and hence lacks intrinsic HS‐degrading activity, the hallmark of heparanase. Hpa2 retains the capacity to bind heparin/HS and exhibits an even higher affinity towards HS than heparanase, thus competing for HS binding and inhibiting heparanase enzymatic activity. It appears that Hpa2 functions as a natural inhibitor of Hpa1 regulates the expression of selected genes that maintain tissue hemostasis and normal function, and plays a protective role against cancer and inflammation, together emphasizing the significance of maintaining a proper balance between Hpa1 and Hpa2.

**Abbreviations**
ECM: extracellular matrix
EGFR: epidermal growth factor receptor
Hpa1: heparinase
Hpa2: heparanase‐2
HS: heparan sulfate
HSPGs: heparan sulfate proteoglycans
VEGF: vascular endothelial growth factor

## BACKGROUND

The heparanase *(HPSE)* messenger RNA (mRNA) encodes a 65 kDa proenzyme that is cleaved by cathepsin L into 8 and 50 kDa subunits that noncovalently associate to form the active enzyme. Structurally, heparanase is composed of a TIM‐barrel fold that contains the enzyme's active site and a flexible C‐terminus domain required for the secretion and signaling function of the protein.^1^, ^2^ Heparanase belongs to the wider class of enzymes known as ‘retaining glycosidases,’ which catalyze hydrolytic cleavage of glycosidic bonds with net retention of anomeric stereochemistry.^3^ It employs a conserved ‘double displacement mechanism,’ involving two key catalytic amino acid residues—a nucleophile (Glu343) and a general acid/base proton donor (Glu225)—and transient formation of a covalent enzyme–substrate intermediate during the catalytic cycle.^3^ The heparin/heparan sulfate (HS)‐binding domains (HBD1, HBD2) are situated close to the active site micropocked fold.^1^, ^3^ Relevant observations are referred to in Table 1.

**Table 1:** **Key observations**.

*Selected key observations (Heparanase)*
*1975—Endoglucuronidase is responsible for cleavage of heparin by mastocytoma cells*.^4^, ^5^
1982—Purification and characterization of HS‐degrading endoglycosidase in platelets.^6^, ^7^
1983—HS‐degrading endoglycosidase is associated with cancer metastasis.^8^, ^9^
1983—Present. Heparanase promotes the pathogenesis of solid and hematological malignancies.^10^, ^11^, ^12^, ^13^
1983—Present. High levels of heparanase correlate with decreased survival of cancer patients.^14^, ^15^, ^16^, ^17^, ^18^, ^19^, ^20^, ^21^, ^22^, ^23^
1984—Activated T lymphocytes produce a matrix‐degrading HS endoglycosidase.^24^
1987–1989—Experimental metastasis and autoimmunity are attenuated by heparanase‐inhibiting species of heparin.^25^, ^26^, ^27^, ^28^
1986—Heparanase regulates the bioavailability of HS‐bound growth factors, chemokines, and cytokines.^29^, ^30^, ^31^
1999—Heparanase (*HPSE*) gene cloning, expression, and function in tumor progression.^32^, ^33^, ^34^, ^35^, ^36^
1999–2003—Latent heparanase is a heterodimer composed of 8 and 50 kDa subunits connected by a 6 kDa linker peptide.^37^, ^38^, ^39^, ^40^
1999—PI‐88 (= Muparfostat), SST0001 (= Roneparstat), and PG545 (= Pixatomid) attenuate tumor growth and angiogenesis in preclinical models.^41^, ^42^, ^43^, ^44^
2000—Heparanase catalytic mechanism involves a proton donor (Glu‐225) and nucleophile (Glu‐343).^1^
2002—Transcriptional activity of the *HPSE* gene promoter.^45^, ^46^, ^47^, ^48^, ^49^, ^50^
2002—*HPSE* gene silencing inhibits tumor angiogenesis and metastasis.^51^, ^52^
2004—The two subunits of the latent enzyme are connected by a 6 kDa linker peptide that obstructs access to the active site.^37^
2004—*HPSE* overexpressing and null mice unravel physiological functions of the enzyme.^53^, ^54^, ^55^
2004—Cellular uptake of heparanase is mediated by cell membrane HS.^56^
2004—Processing and activation of latent heparanase occur in lysosomes (79) and involves cleavage of the linker segment by cathepsin L.^57^, ^58^
2005—Heparanase is involved in the pathogenesis of sepsis, amyloidosis, colitis, pancreatitis, and tissue fibrosis^59^, ^60^, ^61^, ^62^, ^63^
2005—Identification of heparin/HS‐binding domains of heparanase.^64^
2006—Heparanase regulates VEGF gene expression via activation of Src family members.^65^
2006—Heparanase is involved in coagulation and the pathogenesis of thrombosis and atherosclerosis.^66^, ^67^, ^68^, ^69^, ^70^, ^71^
2009—Heparanase signaling is mediated by its carboxy‐terminal domain.^72^
2011—Nuclear heparanase enhances histone acetyltransferase activity and promotes gene expression.^73^
2012—Heparanase is involved in the pathogenesis of diabetes, diabetic nephropathy, diabetic cardiomyopathy, and kidney dysfunction.^74^, ^75^, ^76^, ^77^, ^78^
2012—Nuclear heparanase interacts with key chromatin‐modifying enzymes and regulates gene transcription.^79^
2012—Heparanase augments Akt, EGFR, and STAT phosphorylation.^80^, ^81^
2013—Heparanase regulates secretion, composition, and function of exosomes.^82^, ^83^, ^84^, ^85^, ^86^
2014—Crystallization, structural characterization, and substrate recognition of human heparanase.^3^, ^87^
2014—Heparanase mediates the crosstalk between cells and the tumor microenvironment.^10^, ^11^, ^12^, ^13^, ^88^
2014—Heparanase is a key mediator of macrophage activation and polarization.^89^, ^90^
2014—HS degradation fragments trigger expression of proinflammatory cytokines through TLR‐4 activation.^89^
2014—Present—Examination of antiheparanase therapies in clinical trials.^41^, ^91^, ^92^, ^93^, ^94^
2015—Present. Heparanase is involved in the pathogenesis viral infection.^95^, ^96^, ^97^, ^98^
2015—Heparanase enhances tumorigenicity by promoting autophagy.^99^
2017—H*eparanase plays a critical role in NK‐ and T‐cell recruitment into tumors*.^100^
2021—Heparanase is involved in sensing DNA damage.^96^
*Key observations (Hpa2)*
*2000*—*HPSE2* gene cloning and characterization.^101^
*2010*—*HPSE2* is mutated in urofacial syndrome, a rare congenital disease featuring urological defects and inverted facial expression.^102^, ^103^, ^104^
2010—Hpa2 interacts with HS with high affinity and inhibits heparanase activity.^105^
2015—Overexpression of *HPSE2* attenuates tumor growth, whereas *HPSE2* gene silencing promotes tumorigenesis.^106^, ^107^, ^108^, ^109^, ^110^, ^111^, ^112^, ^113^, ^114^, ^115^
2016—Hpa2 protects against sepsis, endotoxemia, renal inflammation, and Covid‐19.^116^, ^117^, ^118^
2018—Hpa2 functions as a natural inhibitor of Hpa1, emphasizing the significance of a proper Hpa1/Hpa2 ratio in tissue hemostasis.^116^, ^117^, ^118^
2022—Hpa2‐KO homozygosity is embryonic lethal, indicating an essential involvement of Hpa2 in embryonic development (unpublished).
2022—Hpa2‐KO mice are highly susceptible to aggressive cancer and inflammation (unpublished), emphasizing the protective function of host‐derived Hpa2 and encouraging the development of Hpa2‐based therapy.

Abbreviations: Akt, protein kinase B; EGFR, epidermal growth factor receptor; Hpa2, heparanase‐2; HS, heparan sulfate; KO, knockout; NK, natural killer; STAT, signal transducer and activator of transcription; TLR‐4; toll‐like receptor 4; VEGF, vascular endothelial growth factor.

Heparan sulfate proteoglycans (HSPGs) are a fundamental class of extracellular matrix (ECM) constituents, comprised of pericellular or extracellular core proteins conjugated to one or more chains of the glycosaminoglycan polysaccharide HS.^119^ HSPGs mediate myriad biological processes, including signal transduction, developmental patterning,^120^ cell adhesion,^121^ barrier formation,^122^ endocytosis^123^ and viral entry.^95^, ^124^ These processes largely depend upon the HS polysaccharides adorning the core protein, whose heterogeneous structure allows interaction with multiple partners. Given their heterogeneity and versatility, HSPGs serve as important functional components of the cell surface, glycocalyx, and ECM. Hence, cleavage of HS by heparanase affects a diverse and expanding repertoire of physiological and pathological processes. The enzyme appears to fulfill some normal functions, associated, for example, with vesicular traffic, lysosomal‐based secretion, autophagy, tissue remodeling, HS turnover, and gene transcription.^10^, ^11^, ^12^, ^13^ It activates cells of the innate immune system, promotes the formation of exosomes and autophagosomes, and stimulates signal transduction pathways via enzymatic and nonenzymatic activities.^10^, ^11^, ^12^, ^13^, ^82^ These effects dynamically impact multiple regulatory pathways that together drive tumor growth, dissemination, and drug resistance as well as inflammatory responses.^2^, ^11^, ^12^, ^13^ A key venue by which heparanase accomplishes its multiple effects on cells and tissues is by regulating the bioavailability of HS‐bound growth factors, chemokines, and cytokines. In this way, heparanase mediates tumor‐host crosstalk and promotes basic cellular processes that together orchestrate tissue remodeling.^82^ Among the proteins sequestered by the ECM are typical proangiogenic mediators such as platelet‐derived growth factor, hepatocyte growth factor (HGF), basic fibroblast growth factor, heparin‐binding epithelial growth factor, and vascular endothelial growth factor A (VEGF‐A).^29^, ^30^, ^125^, ^126^ Release of these proteins by heparanase contributes to the strong proangiogenic response observed in preclinical models and clinical settings.^13^, ^31^, ^127^, ^128^, ^129^, ^130^

## HISTORY

Activity capable of cleaving macromolecular heparin at a limited number of sites was first reported in *mastocytoma cells*.^4^ Soon thereafter, Höök et al.^131^ reported an endoglycosidase activity that degrades HS glycosaminoglycans into oligosaccharides. Attempts to purify the enzyme yielded some misleading results, culminating, nearly 10 years later, in purification of the platelet enzyme^7^ and cloning of a single human heparanase complementary DNA sequence, independently by four groups.^32^, ^33^, ^34^, ^35^ Given the structural role of HSPGs in the assembly of the ECM and basement membrane, it was hypothesized that HS‐degrading activity will loosen the ECM, thus promoting cell dissemination. Indeed, early on, heparanase activity was found to correlate with the metastatic potential of tumor cells,^8^, ^9^, ^132^ a correlation that still directs and guides heparanase research. Studies performed before cloning of the *HPSE* gene contributed immensely to our understanding of key features in the biology of the enzyme, its mode of action, and involvement in cancer metastasis and inflammation.^133^, ^134^ Soon after cloning of the *HPSE* gene and the development of antiheparanase antibodies and probes, many studies examined its expression in human tumors compared with the adjacent normal tissue. Immunohistochemistry, in situ hybridization, real‐time‐polymerase chain reaction and enzymatic activity analyses revealed that heparanase is upregulated in essentially all human tumors examined.^2^, ^11^, ^12^, ^13^, ^14^, ^128^, ^130^, ^135^ In contrast, the normal‐looking tissue adjacent to the malignant lesion expresses little or no detectable levels of heparanase, indicating that fibroblasts and epithelial cells do not normally express the enzyme. The molecular mechanisms underlying heparanase induction in tumor cells are not entirely clear but involve epigenetic alterations (i.e., DNA methylation), hormones, oncogenes, and transcriptional/posttranscriptional regulation by elements (3ʹ‐UTR, enhancer, insulator) that activate or suppress the *HPSE* promoter.^12^, ^45^, ^136^ Selected observations are referred to in Table 1.

Clinically, patients that were diagnosed as heparanase‐positive exhibited a significantly higher rate of local and distant metastases as well as reduced postoperative survival, compared with patients that were diagnosed as heparanase‐negative.^15^, ^16^, ^17^, ^137^ These and more recent studies^18^, ^19^, ^20^, ^21^, ^22^, ^23^ provide strong clinical support for the prometastatic function of heparanase. Subsequent studies provided compelling evidence that ties heparanase levels with all steps of tumor formation including tumor initiation, angiogenesis, growth, metastasis, and chemoresistance.^22^, ^88^, ^99^, ^138^, ^139^, ^140^, ^141^ Heparanase not only enhances tumor cell dissemination but also accelerates the growth of the primary tumor. We and others have shown that heparanase induces the expression of VEGF‐A^65^ and VEGF‐C,^142^, ^143^ leading to increased blood and lymph vessel density. Subsequent studies revealed that heparanase downregulates the expression of tumor suppressors (i.e., CXCL10^139^) and induces the transcription of proangiogenic (i.e., cyclooxygenase–2, matrix metalloproteinase–9), prothrombotic (i.e., tissue factor [TF]), proinflammatory (i.e., tumor necrosis factor‐α, interleukin [IL]‐1, IL‐6, macrophage inflammatory protein–2), profibrotic (i.e., transforming growth factor‐β), mitogenic (i.e., HGF), osteolytic (receptor activator of nuclear factor kappa beta) and other genes,^66^, ^89^, ^90^, ^128^, ^142^, ^144^, ^145^, ^146^ thus significantly expanding its functional repertoire and mode of action in promoting tissue inflammation and aggressive tumor behavior.^96^ Several excellent up‐to‐date reviews describe basic and translational aspects of heparanase.^11^, ^12^, ^96^ This review is aimed at further increasing awareness of this multifaceted protein, highlighting the significance of the Hpa1–heparanase‐2 (Hpa2) axis and addressing obstacles in implementing antiheparanase therapies.

## NONENZYMATIC ACTIVITIES

**Figure 1:**
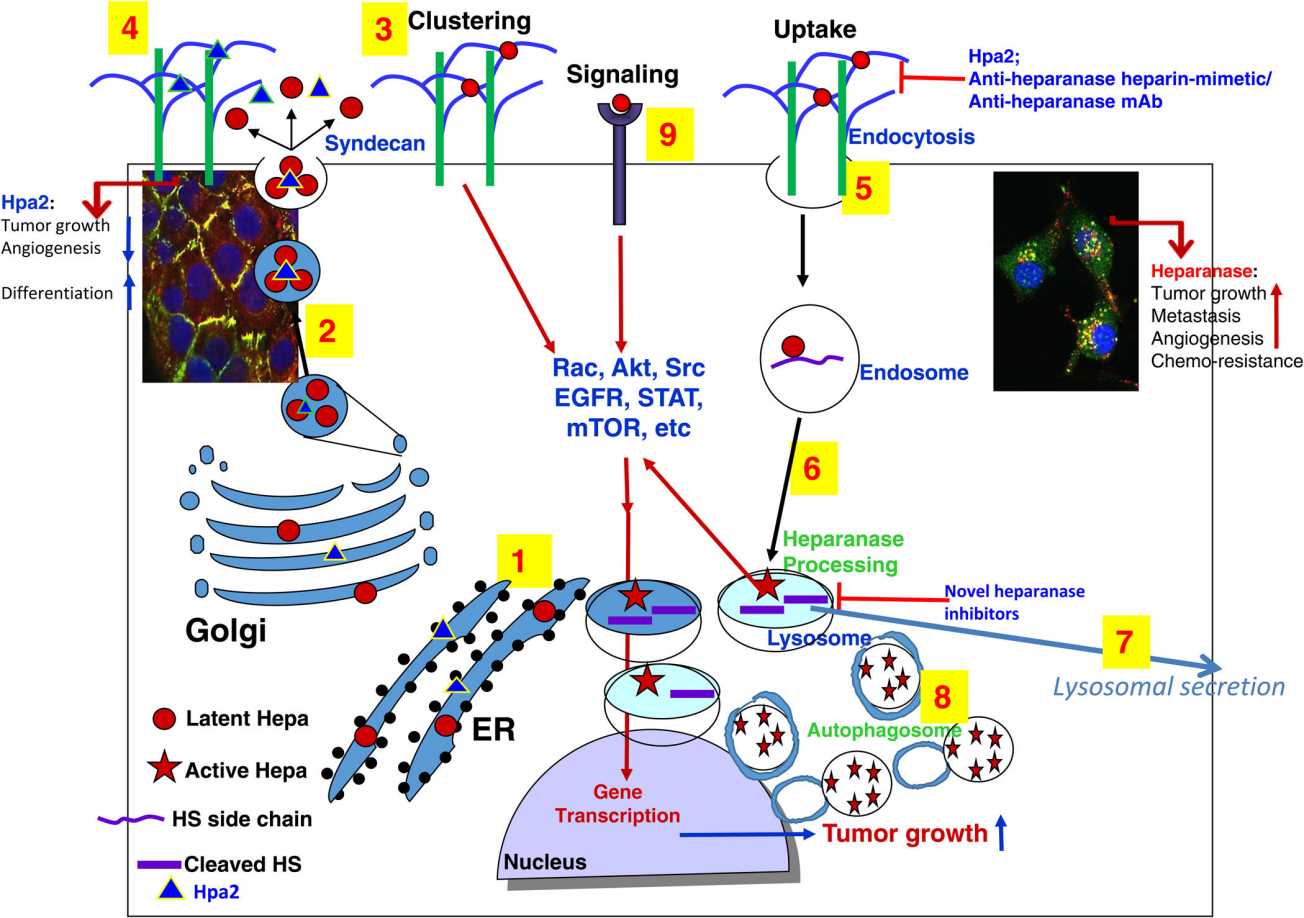
Schematic presentation of heparanase and Hpa2 biosynthesis and trafficking. Pre–proheparanase (red circles) and Hpa2 (blue triangles) are first targeted to the ER lumen via their own signal peptides (1). The proteins are then shuttled to the Golgi apparatus and are subsequently secreted via vesicles that bud from the Golgi (2). Once secreted, heparanase rapidly interacts with syndecans, resulting in their clustering and signaling (3), followed by rapid endocytosis of the heparanase–syndecan complex (5) that accumulates in late endosomes (6). Hpa2 interacts with cell membrane HSPG (i.e., syndecans) with higher affinity but unlike heparanase, is not subjected to uptake but rather remains on the cell membrane for a relatively long period of time (4 and left inset). Accumulation of Hpa2 in the extracellular compartment is enhanced by heparin or anti‐Hpa2 monoclonal antibody. Heparanase uptake is inhibited by heparin/heparin mimetics, antiheparanase monoclonal antibodies, or Hpa2, resulting in extracellular accumulation of the latent enzyme (5). Conversion of endosomes to lysosomes (6) results in heparanase processing and activation (primarily by cathepsin L) awaiting secretion (7). Typically, heparanase appears in perinuclear lysosomes (right inset), promoting autophagy (8) and tumor growth, metastasis, angiogenesis, and chemoresistance due to its enzymatic and signaling (9) functions. Hpa2, on the other hand, attenuates tumor growth and vascularity. Novel heparanase inhibitors are expected to target extracellular latent (signaling) and active heparanase as well as the intracellular, lysosomal, enzyme. ER, endoplasmic reticulum; Hpa2, heparanase‐2; HSPG, heparan sulfate proteoglycan.

Years before the resolution of the heparanase crystal structure, Fux et al.^72^ predicted the structure of enzymatically active, single‐chain, heparanase enzyme, in which the linker segment was replaced by three glycine–serine repeats (GS3), resulting in a constitutively active enzyme.^37^ The structure clearly illustrated a C‐terminus (═C‐domain) fold positioned next to the TIM‐barrel structure.^72^ Remarkably, protein kinase B (Akt) phosphorylation was stimulated by cells overexpressing the C‐domain (amino acids 413–543), while the TIM‐barrel protein variant yielded no Akt activation,^72^ indicating a nonenzymatic signaling function of heparanase mediated by the C‐domain. Notably, Akt phosphorylation was best enhanced in cells transfected with a mini gene comprising a segment of the 8 kDa subunit (Gln^36^–Ser^55^) linked to the C‐domain sequence.^72^ These findings further indicate that the C‐domain is indeed a valid functional domain responsible for Akt phosphorylation. The cellular consequences of C‐domain overexpression were best revealed by monitoring tumor xenograft growth. Notably, tumor xenografts produced by C‐domain‐transfected glioma cells appeared comparable to those produced by cells transfected with the full‐length heparanase.^72^ While signaling through HS clustering appears straightforward in its rationale, HS‐independent signaling by heparanase requires a mediator, possibly in the form of cell surface receptor(s). Binding studies performed with wild‐type CHO‐KI cells and their HS‐deficient CHO‐745 counterpart cells reinforced the notion that while HSPGs serve as low affinity, high abundant binding sites, heparanase also associates with high affinity, low abundant cell surface receptor(s).^72^ Notably, Wood and Hulett^147^ have reported that the 300 kDa cation‐independent mannose 6‐phosphate receptor (CD222) can bind enzymatically active heparanase and may serve as a heparanase receptor (Figure 1).

Another way used to distinguish between enzymatic and nonenzymatic functions of heparanase applied a double mutant protein devoid of heparanase enzymatic activity due to point mutations (Glu_225_–Ala = proton donor, Glu_343_–Ala = nucleophile) in the enzyme' active site.^1^ This inactive form of heparanase was found to exert epidermal growth factor receptor (EGFR) phosphorylation and Akt phosphorylation.^80^ It was also reported that enzymatically quiescent heparanase augmented T‐cell interactions with VCAM‐1 and ECM components.^148^ Notably, heparanase enhances platelet adhesive capacity and thrombogenicity^149^ and also supports the clustering of circulating tumor cells^150^ thereby contributing to the metastatic cascade, largely independent of its enzymatic activity. Likewise, the upregulation of HGF, MCP‐1, and TF expression in response to heparanase was independent of its enzyme activity.^145^, ^151^, ^152^ Moreover, heparanase enhances the phosphorylation of selected signaling molecules including Akt, Src, and EGFR, which in turn facilitates STAT3 phosphorylation, in a manner that requires secretion but not enzymatic activity of heparanase, evident by being mediated by the enzyme C‐terminus domain and by the inactive double mutant protein^72^, ^80^, ^81^ (Table 1). A later observation of integrin‐dependent phosphoinositide 3‐kinase (PI3K)/Akt activation in response to heparanase further highlighted the nonenzymatic activity of heparanase in promoting signal transduction.^153^ Notably, the ability of heparanase to activate PI3K/Akt in a nonenzymatic manner, essentially bypassing PTEN signaling, is evidence of its ability to counter tumor‐suppressive mechanisms.^11^, ^153^ The results suggest that in certain cells, heparanase activates SRC family kinase in an enzymatically independent manner which proceeds to stimulate EGFR. EGFR can then activate downstream pathways such as PI3K–Akt and mitogen‐activated protein kinase–extracellular signal‐regulated kinase, possibly resulting in phosphorylation of signal transducer and activator of transcription 3 (STAT3) which then drives tumorigenic responses.^10^, ^128^

## SIGNAL TRANSDUCTION

Heparanase interacts with syndecans by virtue of their HS content and the typical high affinity that exists between the enzyme and its substrate. This high‐affinity interaction directs the clustering of syndecans followed by rapid and efficient uptake of heparanase^56^, ^154^ (Figure 1). Mechanistically, syndecan clustering by heparanase or the KKDC peptide, corresponding to the heparin‐binding domain of heparanase,^64^ enhance cell adhesion and spreading, associated with PKC, Src, and Rac1 activation,^155^ molecular determinants shown to be induced by syndecans.^156^, ^157^, ^158^ Cell adhesion represents a nonenzymatic signaling function of heparanase in its simplest term.^159^, ^160^ Heparanase was also noted to elicit signaling in a manner that does not involve HS. Signaling is considered to be HS‐independent if it occurs in HS‐deficient cells (i.e., CHO‐745) or in the presence of heparin, as demonstrated for enhanced Akt phosphorylation by heparanase.^161^ Heparin, a potent competitive inhibitor of heparanase enzymatic activity, when added together with heparanase, augmented, rather than attenuated, Akt phosphorylation,^161^ critically implying that heparanase enzymatic activity is not required for Akt activation. Importantly, in xenograft models, heparanase overexpression resulted in tumors bigger in size^45^, ^162^ coupled with increased Akt phosphorylation.^45^, ^72^ Adversely, heparanase gene silencing was associated with reduced Akt phosphorylation.^140^, ^163^ Related studies revealed that heparanase stimulates the phosphorylation of STAT3, STAT5, Src, EGFR, Erk, and the insulin receptor and also activates G‐protein receptor signaling,^65^, ^80^, ^81^, ^164^ all function to promote tumorigenesis (Figure 1 and Table 1).

## DNA DAMAGE

Associations between heparanase and various pathologies, including inflammation and cancer metastasis, have historically been attributed to the cleavage of HS chains at the cell surface and basement membrane; however, as discussed above, heparanase may possess unique roles arising independently of its enzymatic active site. For example, while the enzymatic activity of heparanase appears to enable viral release through splitting of HS residues at the cell surface, heparanase was reported to also regulate gene expression and trigger proviral signaling through some distinct nonenzymatic activity.^97^ It appears that heparanase acts beyond its established endoglycosidase activity as a potent regulator of the signal transduction phase of cellular defense. In this respect, it was reported that cells lacking heparanase display enhanced sensitivity to DNA damage‐induced death^96^ and are intrinsically resistant to herpes simplex virus‐1 infection.^96^ Moreover, the interferon system is constitutively enhanced in the absence of heparanase and deletion of heparanase protects against cellular infiltration and associated inflammation.^96^, ^97^, ^165^ Repeat immunopurifications of heparanase showed robust binding of proteins heavily implicated in DNA damage sensing and repair, suggesting that heparanase plays a significant role as a regulator of DNA damage response signals.^96^

## NUCLEAR HEPARANASE

Nuclear HS inhibits histone acetyltransferases (HATs), and thereby gene transcription.^73^ Heparanase contains two potential nuclear localization sequences, and enzymatically active heparanase has been found in the chromatin compartment of the nucleus where it colocalizes with RNA polymerase II and positively controls the transcription of genes important for T cells' immune function.^79^ Likewise, by entering the nucleus and degrading nuclear syndecan‐1, heparanase mediates HAT activation and transcription of genes associated with an aggressive tumor phenotype.^73^ Conversely, nuclear heparanase binds nonspecifically to DNA and competes for binding with nuclear factor‐κB (NF‐κB), thus preventing transcription of NF‐κB target genes and acting as a tumor suppressor.^166^ At the molecular level, nuclear heparanase appears, among other effects, to regulate histone 3 lysine 4 methylation by influencing the recruitment of demethylases to transcriptionally active genes.^79^ Together it appears that nuclear heparanase promotes chromatin remodeling that opens its conformation allowing access to promotors of genes that affect cancer progression.^167^, ^168^ In‐depth research is still needed to better elucidate the mode of heparanase nuclear translocation and transcriptional activity.^73^, ^79^

## HEPARANASE‐INHIBITING COMPOUNDS

To date, only four compounds have progressed to clinical trials.^10^, ^41^, ^91^, ^92^, ^93^, ^129^ These four “best‐in‐class” inhibitors are all polyanionic oligo‐/polysaccharides that mimic physicochemical properties of the natural heparanase inhibitor heparin, a glycosaminoglycan related to HS. The heparin‐like properties of these inhibitors, along with their structural heterogeneity, likely produce unwanted pleiotropic effects (e.g., anticoagulation, growth factor binding, poor pharmacokinetics) that complicate their clinical use. The active site of heparanase has proven challenging for small‐molecule pharmacological intervention, given its extensive interaction surface evolved to bind large HS polysaccharides.^169^ Such challenging sites are often well targeted by mechanism‐based covalent inhibitors, which may still react with an enzyme despite weak or transient initial binding. Indeed, HS‐configured pseudodisaccharide, designed to bind selectively to the heparanase active site and react with its essential catalytic nucleophile, was recently synthesized.^169^ This nanomolar, mechanism‐based, irreversible heparanase inhibitor markedly reduced cancer aggression in in cellulo and in vivo cancer models.^169^ Recently, chemoenzymatic synthesis of sulfur‐linked sugar polymers was reported to yield potent competitive heparanase inhibitors.^170^ Importantly, heparanase knockout (KO) mice exhibit no obvious immunological and other deficits,^53^ implying that inhibition of heparanase will cause minimal side effects in cancer patients.

Dual and apparently antagonistic effects of antiheparanase therapy should be considered.^11^ For example, it was reported that heparanase plays a critical role in natural killer (NK) cell invasion into tumors.^100^ It was shown that cytokine and immune checkpoint blockade immunotherapy for metastases were compromised when NK cells lacked heparanase.^100^ Likewise, it was found that in contrast to freshly isolated T lymphocytes, *HPSE* mRNA is downregulated in in vitro‐expanded T cells. This may explain the reduced ability of cultured CAR‐T cells to penetrate stroma‐rich solid tumors compared with lymphoid tissues. Indeed, engineering the CAR‐T cells to express *HPSE* resulted in their improved capacity to degrade the ECM, which promoted tumor T cell infiltration and antitumor activity.^171^ It was suggested that the use of this strategy might enhance the activity of CAR‐T cells in individuals with stroma‐rich solid tumors. These results should be considered when systemically treating cancer patients with heparanase inhibitors since the potential adverse effect on NK and CAR‐T cells on cell infiltration might limit the antitumor activity of the inhibitors.^171^ It appears that targeting the tumor microenvironment by heparanase inhibitors enhances the antitumor activity of approved therapies, further providing a strong rationale for applying antiheparanase therapy in combination with conventional anticancer drugs.^141^ Remarkably, heparanase inhibitors were effective even when the xenografted tumor cells were devoid of heparanase, emphasizing the significance of heparanase contributed by cells residing in the tumor microenvironment.^88^

## HPA2—A NEW PLAYER IN THE HEPARANASE FIELD

**Figure 2:**
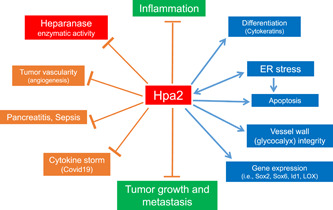
Protective effects of Hpa2 against cancer, inflammation, and tissue damage. Hpa2 functions in heparanase‐dependent and independent manners, and affects gene expression (i.e., Sox2, Id1, Lox), tumor vascularity, ER stress, and cell apoptosis, resulting in cancer suppression.^2^, ^106^, ^107^, ^114^, ^129^ Hpa2 levels are elevated in response to stress conditions, generating a vicious cycle that contributes to attenuation of tumor progression. Hpa2 exerts a protective effect in models of sepsis, endotoxemia, diabetes‐induced renal inflammation, pancreatitis, and in patients with Covid‐19, attributed, in part, to inhibition of heparanase‐mediated damage to the integrity of the glycocalyx.^116^, ^117^, ^118^ Covid‐19, coronavirus disease 2019; ER, endoplasmic reticulum; Hpa2, heparanase‐2.

*HPSE2*, the gene encoding Hpa2, was cloned soon after the cloning of heparanase, based on sequence homology.^101^ Hpa2 gained attention when it was found that the HPSE2 gene is mutated in a human disease called urofacial syndrome (UFS).^102^, ^103^ UFS is a rare autosomal recessive congenital disease, featuring a combination of urological defects and an inverted facial expression attributed to peripheral neuropathy.^104^ Notably, Hpa2 lacks intrinsic HS‐degrading activity, the hallmark of heparanase,^105^ yet it retains the capacity to interact with HS.^105^ Hpa2 exhibits an even higher affinity towards heparin and HS than heparanase,^105^ thus competing for HS binding and thereby inhibiting heparanase enzymatic activity.^105^ Exogenously added Hpa2 binds to HS and clusters syndecan‐1 and 4. Surprisingly, however, unlike heparanase, it fails to get internalized and remains on the cell surface^105^ (Figure 2). Hpa2 regulates selected genes that promote normal differentiation, endoplasmic reticulum (ER) stress, fibrosis, and apoptosis, resulting in antitumor, antiangiogenic, and anti‐inflammatory effects.^2^, ^10^, ^106^, ^116^, ^129^ Interestingly, stress conditions induce the expression of Hpa2, thus establishing a feedback loop by which Hpa2 enhances ER stress which, in turn, induces Hpa2 expression^107^, ^108^, ^109^ (Figure 2). Unlike the intense research effort devoted to exploring the significance of heparanase in cancer progression, very little attention was given to Hpa2. Recent evidence indicates, nonetheless, that Hpa2 expressed by both the tumor cells and the host tumor microenvironment functions as a tumor suppressor. Clinically, it was reported that, unlike heparanase, Hpa2 expression is readily detected in normal epithelium of the bladder, breast, cervical, gastric, and ovarian tissues, whereas its expression is substantially decreased in the resulting carcinomas,^10^, ^108^, ^109^, ^110^, ^111^, ^112^ expression pattern that is typical of a tumor suppressor. Furthermore, patients that retain high levels of Hpa2 survived longer than patients bearing Hpa2‐low tumors.^105^, ^107^, ^108^, ^109^, ^113^ Experimentally, overexpression of Hpa2 attenuated the growth of tumor xenografts, whereas Hpa2 gene silencing resulted in bigger tumors.^106^, ^107^, ^108^, ^109^, ^111^, ^114^, ^115^ This is best demonstrated by gene editing of Hpa2 in pharyngeal FaDu cells applying the CRISPR technology. Notably, Hpa2‐null cells produced bigger tumors vs control cells, whereas the rescue of Hpa2 in the null cells resulted in smaller tumors.^114^ Collectively, these results support the notion that Hpa2 functions as a tumor suppressor (Figure 2 and Table 1). Heparanase and Hpa2 not only exhibit opposite functions in terms of tumor growth but also in terms of the underlying mechanism. For example, while heparanase induces VEGF‐A and VEGF‐C^142^ expression and promotes angiogenesis, Hpa2 attenuates the expression of VEGF‐A and VEGF‐C and decreases tumor vascularity^106^; whereas heparanase attenuates cell differentiation and promotes epithelial‐to‐mesenchymal transition (EMT),^172^, ^173^ Hpa2 increases cell differentiation.^106^, ^111^ This mirrored functionality suggests that Hpa2 exerts these properties in part by modulating heparanase, as demonstrated by a significant decrease in heparanase activity in sarcoma cells overexpressing Hpa2.^108^

## CONCLUSIONS AND PERSPECTIVES

Among other aspects, heparanase research reinforced the significance of the ECM in the control of cell proliferation and differentiation.^174^, ^175^ It led to important and often unexpected observations in diverse normal and pathological processes including, wound healing,^176^ angiogenesis,^31^ autophagy,^99^ signal transduction,^72^, ^97^, ^128^, ^153^ protein trafficking,^177^ lysosomal secretion,^129^, ^178^ blood coagulation,^67^, ^151^ EMT,^173^, ^179^ glycocalyx remodeling,^116^, ^180^ activation of immune cells,^24^, ^59^, ^89^, ^90^ exosome formation,^82^, ^83^, ^84^ drug resistance,^13^, ^181^ gene transcription,^73^, ^74^ and other key biological features.^11^, ^12^, ^97^ While most studies emphasize the involvement of heparanase in cancer and inflammation, other pathologies were investigated. Among these are diabetes,^74^, ^75^ diabetic complications (i.e., diabetic nephropathy, diabetic cardiomyopathy),^76^, ^77^ kidney dysfunction,^182^ fibrosis,^78^, ^183^ inflammatory disorders (i.e., neuroinflammation, pancreatitis, ulcerative colitis, arthritis, sepsis),^59^, ^60^, ^184^, ^185^ amyloidosis,^61^ atherosclerosis,^66^, ^68^, ^69^, ^70^ viral diseases,^96^, ^97^ and other pathologies.^11^, ^12^, ^97^ Heparanase accomplishes all these by exerting both enzymatic and nonenzymatic functions that are mostly HS‐dependent yet in some cases are HS‐independent.^10^, ^161^ The enzyme is expressed by tumor cells and cells of the tumor microenvironment and functions extracellularly to remodel the ECM and regulate the bioavailability of HS‐bound factors, as well as intracellularly (i.e., lysosome, nucleus)^10^ (Figure 1). Unraveling these and other aspects of heparanase biology (e.g., mode of heparanase nuclear translocation and transcriptional activity, activation and polarization of macrophages,^59^, ^73^, ^79^, ^89^, ^90^, ^186^ is ongoing and is critical to our understanding of its multiple functions in health and disease.

Unfortunately, antiheparanase‐based therapy has not yet been implemented in the clinic. Notably, all antiheparanase compounds that were or are being examined in clinical trials are heparin/HS‐like saccharides.^10^, ^169^ While these compounds were very successful in numerous mouse models,^18^, ^141^, ^163^ unforeseen adverse effects are documented and ascribed primarily to the poor pharmacokinetics, heterogeneous nature, nonspecific, and pleiotropic effects of those HS mimetics. Development of more specific oligosaccharides, small molecules and neutralizing monoclonal antibodies is ongoing.^88^, ^169^, ^170^, ^187^ Of the four compounds examined in clinical trials, PG545 (Pixatimod) appears the most potent and promising, likely due to its lipophilic moiety and superior pharmacokinetic properties.^92^ The specificity of this compound is, nonetheless, questionable, because it exerts also heparanase‐independent functions and attenuates the growth of tumor xenografts produced by heparanase‐negative lymphoma cells.^188^ Second‐generation heparanase inhibitors, possibly in the form of disaccharides that covalently bind to the enzyme active site,^169^ should target also its intracellular activities and hence better neutralize all aspects of heparanase function. Heparanase is a multifaceted protein having both enzymatic and nonenzymatic activities. To the best of our knowledge, all heparanase inhibitors under development are predominately targeting the enzymatic activity of heparanase. Therefore, a main question raised in the development of antiheparanase inhibitors is whether the enzymatic activity of heparanase is the critical determinant of its protumorigenic and prometastasis effects, given the fact that species of heparanase (i.e., C‐terminus domain, active site double‐mutant, T5 splice variant) lacking enzymatic activity still promotes tumor progression.^10^, ^72^, ^189^, ^190^

Based on the housekeeping nature of its gene promoter, we suggest that heparanase is expressed at low levels by all cells, modulating autophagy and possibly other functions of the lysosome. According to this notion, heparanase function in the lysosome may not be less important than its function extracellularly. This may turn relevant in platelets, neutrophils, lymphocytes, and macrophages that show relatively high levels of heparanase expression and activity.^11^, ^12^, ^33^, ^90^, ^191^ Beyond serving as a cellular recycling center, recent evidence suggests that the lysosome is involved in homeostasis, generating building blocks for cell growth, mitogenic signaling, angiogenesis, metastasis, and activation of transcriptional programs,^192^, ^193^ repertoire that closely resembles those of heparanase. The PI3‐kinase/Akt/mammalian target of rapamycin (mTOR) is highly implicated in the regulation of cell metabolism, protein homeostasis, and cell growth due, in part, to the localization of mTOR at the lysosome membrane which is required for its activation.^194^, ^195^ Indeed, Akt is the most common kinase activated by heparanase,^72^, ^80^, ^140^, ^153^, ^161^, ^163^ and its instrumental role in the regulation of mTOR would likely convey to the lysosome.^194^, ^195^ Research is needed to further resolve the significance of heparanase in modulating lysosomal function in normal and diseased cells.

Unlike heparanase, Hpa2 does not undergo proteolytic processing and hence lacks intrinsic HS‐degrading activity, the hallmark of heparanase. Our working hypothesis is that Hpa2 functions as a natural inhibitor of heparanase, playing a protective role in maintaining tissue hemostasis and normal function and regulating to a large extent the balance between disease progression and suppression (Figure 2). Hpa2 functions in heparanase activity‐ and HS‐dependent and independent manners, and regulates the expression of selected genes that affect tumor vascularity, tumor fibrosis, cell differentiation and apoptosis, resulting in cancer suppression.^10^, ^129^ A protective effect of Hpa2 was also demonstrated in models of sepsis, endotoxemia, and diabetes‐induced renal inflammation,^116^, ^117^ as well as in patients with Covid‐19^118^ (Figure 2), encouraging the development of Hpa2‐based therapies. This was attributed, in part, to inhibition of Hpa1‐mediated endothelial injury and damage to the glycocalyx integrity, emphasizing the physiological significance of maintaining a proper Hpa1/Hpa2 ratio.^116^ Applying the CRISPR/Cas9 technology we have generated constitutive Hpa2‐KO mice. Unexpectedly, we found that Hpa2‐KO homozygosity is embryonic lethal, indicating an essential involvement of Hpa2 in embryonic development (unpublished). We have recently generated conditional Hpa2‐KO mice and found that these mice are highly susceptible to aggressive cancer and inflammation (unpublished), encouraging further research on the protective function of host‐derived Hpa2.

## AUTHOR CONTRIBUTIONS

**Israel Vlodavsky:** Conceptualization; writing—review & editing; supervision; funding acquisition. **Yasmin Kayal:** Investigation. **Maram Hilwi:** Investigation. **Soaad Soboh**: Investigation. **Ralph D. Sanderson:** Writing—review & editing; conceptualization; funding acquisition. **Neta Ilan:** Writing—review & editing; supervision; conceptualization.

## CONFLICT OF INTEREST STATEMENT

The authors declare no conflict of interest

## ETHICS STATEMENT

Not applicable.

## Data Availability

Data sharing is not applicable to this article as no new data were created or analyzed in this study.
